# A novel risk model construction and immune landscape analysis of gastric cancer based on cuproptosis-related long noncoding RNAs

**DOI:** 10.3389/fonc.2022.1015235

**Published:** 2022-10-26

**Authors:** Yuanhang Wang, Kanghui Liu, Kuan Shen, Jian Xiao, Xinyi Zhou, Quan Cheng, Li Hu, Hao Fan, Peidong Ni, Zekuan Xu, Diancai Zhang, Li Yang

**Affiliations:** ^1^ Department of General Surgery, the First Affiliated Hospital of Nanjing Medical University, Nanjing, China; ^2^ Department of General Surgery, Liyang People’s Hospital, Liyang Branch Hospital of Jiangsu Province Hospital, Liyang, China

**Keywords:** cuproptosis-related lncRNA, gastric cancer, prognosis, tumor immune microenvironment, immunotherapy, risk model

## Abstract

Recent studies have identified cuproptosis, a new mechanism of regulating cell death. Accumulating evidence suggests that copper homeostasis is associated with tumorigenesis and tumor progression, however, the clinical significance of cuproptosis in gastric cancer (GC) is unclear. In this study, we obtained 26 prognostic cuproptosis-related lncRNAs (CRLs) based on 19 cuproptosis-related genes (CRGs) *via* Pearson correlation analysis, differential expression analysis, and univariate Cox analysis. A risk model based on 10 CRLs was established with the least absolute shrinkage and selection operator (LASSO) Cox regression analysis and multivariate Cox proportional hazards model to predict the prognosis and immune landscape of GC patients from The Cancer Genome Atlas (TCGA). The risk model has excellent accuracy and efficiency in predicting prognosis of GC patients (Area Under Curve (AUC) = 0.742, 0.803, 0.806 at 1,3,5 years, respectively, *P < 0.05*). In addition, we found that the risk score was negatively correlated with the infiltration of natural killer (NK) cells and helper T cells, while positively correlated with the infiltration of monocytes, macrophages, mast cells, and neutrophils. Moreover, we evaluated the difference in drug sensitivity of patients with different risk patterns. Furthermore, low-risk patients showed higher tumor mutation burden (TMB) and better immunotherapy response than high-risk patients. In the end, we confirmed the oncogenic role of AL121748.1 which exhibited the highest Hazard Ratio (HR) value among 10 CRLs in GC *via* cellular functional experiments. In conclusion, our risk model shows a significant role in tumor immunity and could be applied to predict the prognosis of GC patients.

## Introduction

Gastric cancer (GC) remains one of the most common malignant tumors of the gastrointestinal tract, with more than one million new cases and an estimated 0.76 million new deaths each year globally. Despite improvements in the diagnosis and treatment of GC, the overall survival of patients with advanced gastric cancer (AGC) remains poor (1). HER2, microsatellite instability (MSI), and PD-L1 are only three targeted therapy biomarkers that can predict the therapeutic response of GC patients (2). Therefore, it is crucial to identify novel biomarkers that can predict prognosis and therapeutic response in patients with GC.

Long non-coding RNA (LncRNA) is a class of transcripts longer than 200 nucleotides with limited or no protein-coding properties (3, 4). Accumulated evidence suggests that lncRNAs are implicated in regulating various biological processes during tumor development (5). They are critical operators in tumorigenesis, metastasis, progression, and treatment resistance (5–7). Studies have shown that several lncRNAs that are elevated in GC, such as H19, HOXA11-AS, PVT1, and MALAT1, are involved in the tumorigenesis, metastasis, and angiogenesis of GC (8–11). Meanwhile, several downregulated lncRNAs such as TUSC7 and MEG3 were identified as tumor suppressors (12–14). These findings imply the potential use of GC-specific LncRNAs as biomarkers and therapeutic targets.

A recent study found that the accumulation of intracellular copper causes the aggregation of mitochondrial lipoylated proteins and the destabilization of Fe-S cluster proteins, leading to a unique type of cell death termed cuproptosis (15). Cuproptosis is mediated by protein lipoylation, which differs from other mechanisms of regulated cell death, such as ferroptosis, apoptosis, necroptosis, and pyroptosis (16). According to the research conducted by Tsvetkov and colleagues, respiring cells and TCA-cycle active cells had higher quantities of lipoylated tricarboxylic acid cycle (TCA) enzymes, and the lipoyl moiety functions as a direct copper binder, leading to aggregation of the lipoylated protein, Fe-S cluster-containing proteins loss, and HSP70 induction, reflective of acute proteotoxic stress (16). In more detail, in the extracellular environment, elesclomol binds copper (Cu^2+^) and transports it to intracellular compartments. Cuproptosis is primarily brought on by increased Cu concentration through mitochondrial proteotoxic stress, which is mediated by ferredoxin 1 (FDX1). FDX1 facilitates the lipoylation and aggregation of enzymes responsible for the regulation of the mitochondrial TCA cycle by reducing Cu2^+^ to Cu^+^. However, the instability of iron-sulfur proteins (Fe-S) cluster proteins is brought on by FDX1. In addition to Cu ionophores, Cu exporters such as ATPase Copper Transporting Beta (ATP7B) and importers like Solute Carrier Family 31 Member 1 (SLC31A1) control intracellular Cu^+^ levels to regulate cuproptosis sensitivity (15).

In our study, we identified 10 CRLs and established a risk model. We evaluated the prognostic value of the model established in GC patients and compared the predictive prognosis efficiency of the risk model with those of other clinical characteristics. In addition, in GC patients, we explored the association between risk scores and clinical characteristics, immune cell infiltration, immunotherapy score, drug sensitivity, and TMB. Moreover, we confirmed the oncogenic role of lncRNA AL121748.1 in GC *via* CCK-8 assay, colony formation assay, wound healing assay, and transwell assays. This study aimed to find a new biomarker for the clinical prediction of therapeutic response and prognosis in GC.

## Materials and methods

### Data acquisition and processing

From The Cancer Genome Atlas (TCGA), we obtained the expression matrix and corresponding clinical data of GC patients and excluded those with a survival time of fewer than 30 days. A total of 342 patients were randomly assigned to two groups, with 172 patients in the training set and 170 patients in the validation set. To annotate mRNA and lncRNA, we used the annotation human GTF file from Ensembl (http://asia.ensembl.org).

### Obtaining cuproptosis-related lncRNAs

Based on previous research (15, 17, 18), we obtained 19 CRGs and obtained gene expression data *via* the “limma” package in R. We then identified 1218 CRLs based on 19 CRGs from lncRNA expression data *via* Pearson’s correlation analysis (|Pearson ratio| > 0.3, P < 0.001).

### Establishment of the risk model

After acquiring 1218 CRLs, 26 prognostic CRLs were obtained *via* univariate analysis. LASSO regression analysis and multi-cox analysis were then conducted to gain the risk model based on 26 prognostic CRLs. 10 lncRNAs were finally identified for the construction of the model. We calculated the risk score as follows:


$$Riskscore(patients)=\sum_{k=1}^{n}Coefvalue(genek)*expression(genek)$$

The terms n, k, coef, and expression in this formula stand for the numbers of lncRNAs, selected lncRNAs, the value of the regression coefficient, and the value of the lncRNA expression, respectively. The cutoff value was assumed to be the median of the risk score in the training set.

### Validation of the risk model

A Kaplan-Meier analysis was conducted to investigate the differences in patients’ survival between the two groups. Receiver operating characteristic (ROC) curves were used to determine the model’s accuracy. The survival status of patients with various risk patterns was also plotted. In the above analysis, the R packages “timeROC”, “ survminer ”, and “survival” were used. By using the “pheatmap” package in R, the RNA level of 10 lncRNAs in the risk model was visualized. The model’s effectiveness and accuracy were further validated in the testing set.

### Prognostic function of the risk model

The chi-square test was applied to determine the relationship between risk patterns and clinical characteristics of GC patients. To confirm the model’s independent prognostic function, both univariate and multivariate analyses were performed. Clinically relevant ROC curves and decision curves were used to validate the risk model’s clinical application value. A nomogram was used to calculate the predicted survival time of GC patients, and the accuracy of it was determined *via* the calibration and ROC curves. The R packages “rms”, “ggDCA”, “survival”, “ replot ”, and “ timeROC” were used in the above analysis. The difference in overall survival (OS) among patients with various clinicopathological characteristics was evaluated *via* Kaplan-Meier survival analysis.

### Immune cells infiltration in GC patients

We obtained integrated TCGA immune cell infiltration data (CIBERSORT, TIMER, QUANTISEQ, XCELL, EPIC, and MCPCOUNTER) from TIMER2.0 (https://timer.comp-genomics.org). Spearman correlation analysis was used to determine the relationship between immune infiltrating cells and risk score. The R packages “limma”, “ ggplot2”, “scales”, “ pheatmap”, “ ggpubr”, “reshape2”, “tidyverse”, and “ ggtext ” were used in the above analyses.

### Therapeutic sensitivity prediction of patients with different risk patterns

Using the pRophetic algorithm, we evaluated the 50 percent inhibiting concentration (IC50) of drugs to determine the value of the risk score in predicting chemotherapies/targeted drug sensitivity. The drugs with p < 0.001 were displayed. We gained the patients’ immunotherapy score data from (http://tide.dfci.harvard.edu/) and the therapeutic sensitivity to immunotherapy of patients in different risk groups was evaluated. The R packages “limma”, “ ggpubr ”, “reshape2”, and “ ggplot2” were used in the above analysis.

### Relationship between the risk model and tumor mutation burden

We obtained tumor mutational burden (TMB) data from the TCGA database. The relationship between TMB and risk score was then evaluated and visualized *via* the R packages “ggpubr”, “ggplot2”, and “reshape2”. The difference in TMB level was visualized *via* the R package “maftools”. The difference in survival between patients with various patterns of TMB and risk was evaluated *via* Kaplan-Meier analysis.

### Statistical analysis

All bioinformatic results were obtained and generated by using Perl (5.32.1.1) or R (version 4.1.2). Each section’s statistical methods were described above. Other materials and methods used in this study can be seen in **Supplementary Material and Methods**.

## Results

### Landscape of cuproptosis-related genes in GC patients

**Figure 1:**
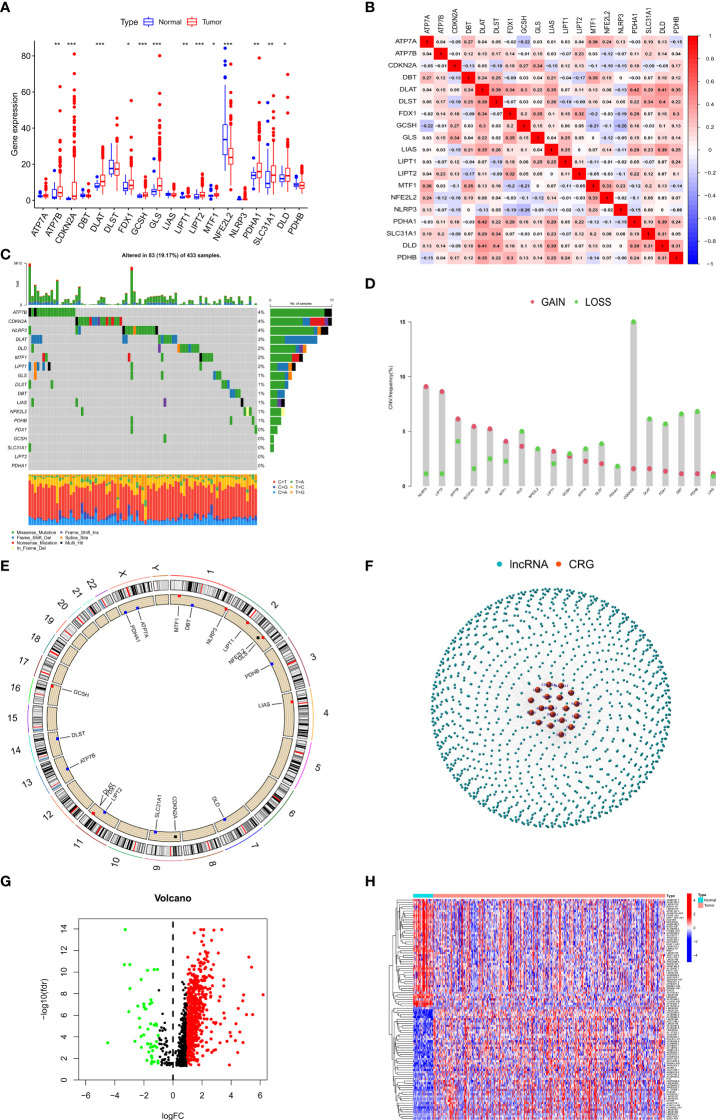
Landscape of CRGs and identification of CRLs in GC patients. **(A)** The expression of 19 CRGs in GC and normal tissues. **(B)** Correlation analysis of 19 CRGs. **(C)** The mutation frequency of 19 CRGs in STAD. **(D)** The CNV variation frequency of 19 CRGs in STAD. **(E)** The location of CNV alteration of 19 CRGs on 23 chromosomes in STAD. **(F)** Gene coexpression network map of CRGs and lncRNAs. **(G)** Volcano plot shows differentially expressed CRLs. **(H)** Heatmap showed the top 50 differentially expressed CRLs. *p < 0.05, **p < 0.01, and ***p < 0.001.

We first evaluated the differences in the expression of 19 CRGs between GC patients and normal individuals. Interestingly, the boxplot showed that 13 of 19 CRGs were aberrantly expressed in GC patients (**Figure 1A**). In addition, we investigated the association between the 19 CRGs and showed that DLAT and PDHA1 had the highest positive correlation (r=0.42) while NLRP3 and GCSH had the highest negative correlation (r=0.26) (**Figure 1B**). We also explored the mutation and copy number variation (CNV) of 19 CRGs in GC patients, showing that mutations were present in 83/433 GC patients, missense mutation was the most common form of mutation in CRGs, and the ATP7B had the highest mutation rate of about 4% among the 19 CRGs (**Figure 1C**). We then explored the copy number variation of the 19 CRGs and showed that copy number deletions were present in all CRGs, with CDKN2A having the highest copy number deletion of about 15%, similarly, copy number amplifications were also present in all CRGs, with NLRP3 having the highest copy number amplification of about 9% (**Figures 1D, E**).

Considering the important role of lncRNAs in regulating gastric carcinogenesis and progression, we conducted a Pearson correlation analysis (|cor| > 0.3, *p* < 0.001) on 14086 lncRNAs and 19 CRGs to screen out 1218 CRLs (**Figure1F**), and we subsequently performed differential analysis (|logFC| > 1, FDR < 0.05) on these 1218 lncRNAs to screen out 687 differentially expressed lncRNAs for subsequent analysis (**Figures 1G, H**).

### Construction of the risk model

**Figure 2:**
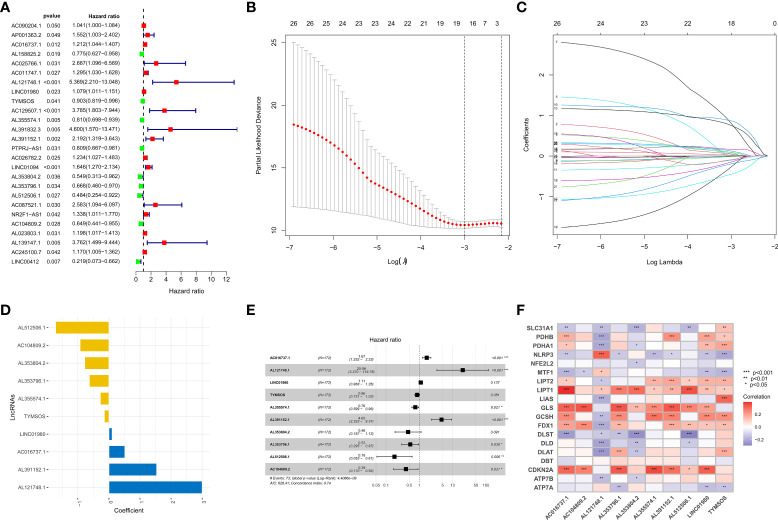
Establishment of the risk model. **(A)** Forest plot of 26 prognosis-related lncRNAs after univariate Cox regression analysis (p < 0.05). **(B, C)** LASSO regression of 26 prognosis-related lncRNAs. **(D, E)** The HR and coefficient of 10 CRLs involved in the multivariate Cox proportional hazards model. **(F)** The correlation between 19 CRGs and 10 CRLs in the risk model was visualized. *p < 0.05, **p < 0.01, and ***p < 0.001.

**Figure 3:**
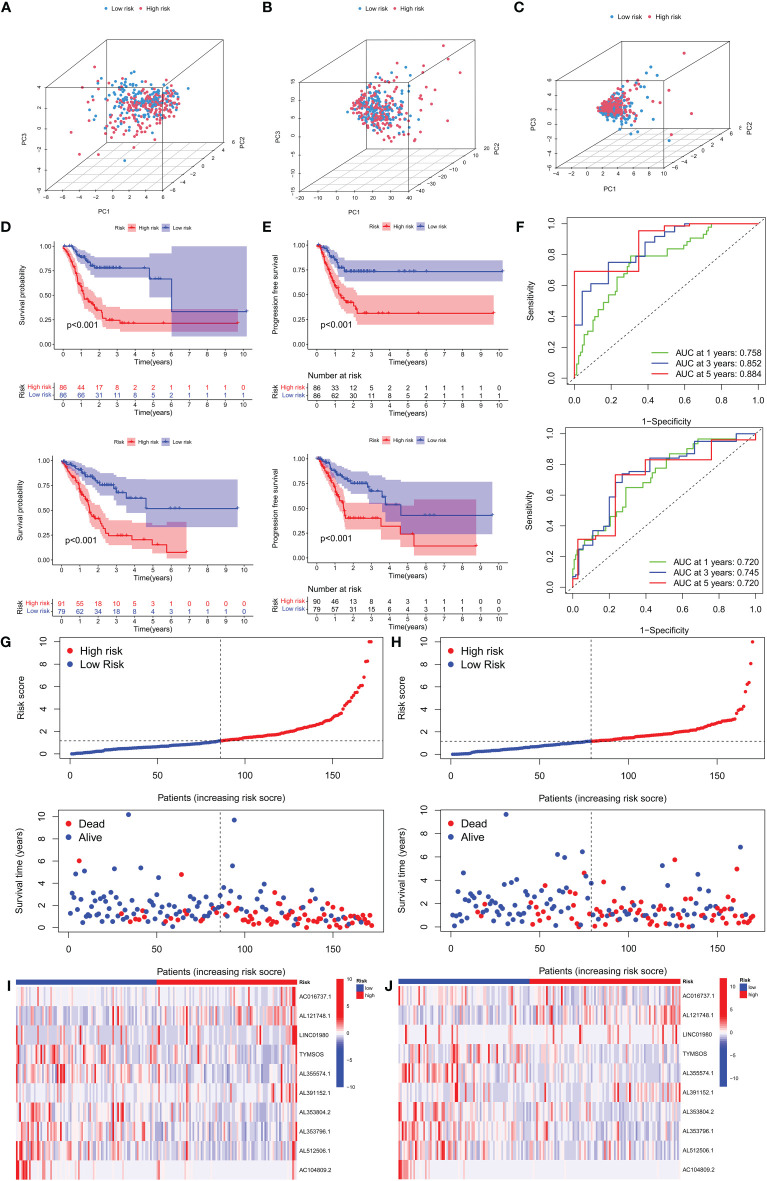
Survival analysis and validation of the risk model in the training set and testing set. **(A)** PCA analysis was conducted for the entire gene set, **(B)** 19 CRGs, and **(C)** 10 CRLs in the risk model. **(D, E)** The survival difference of patients with different risk patterns in the training set and testing set. **(F)** A time-dependent ROC curve was plotted to test the accuracy of the risk model. **(G, H)** Patients in the training set and testing set were ranked according to the risk score. Then, the survival status difference of the patients between the two groups was visualized. **(I, J)** Heatmap showed the RNA level of the 10 CRLs in the training set and testing set.

To identify prognosis-related lncRNAs, the survival data of GC patients were combined with the expression data of lncRNAs, and we subsequently obtained 26 prognosis-related lncRNAs *via* univariate analysis (**Figure 2A**). We excluded patients with a survival time of fewer than 30 days or no survival data. Then 342 GC patients were randomly assigned to two groups (training set or testing set), and there were no statistical differences in the clinical characteristics of all patients between the two sets (**Supplementary Table 1**). To reduce the number of genes for constructing the model, we performed a lasso regression analysis to obtain 18 modeled prognosis-related lncRNAs (**Figures 2B, C**). Subsequently, we performed a multivariate regression analysis on these 18 lncRNAs and screened 10 lncRNAs for the construction of the risk model finally (**Figures 2D, E** and **Supplementary Table 2**). The relationship between 10 lncRNAs in the model and CRGs was visualized (**Figure 2F**). The risk score of each patient was calculated according to the formula mentioned previously. We performed a principal component analysis (PCA) based on the risk pattern of each patient to confirm whether the established risk model could distinguish between high-risk and low-risk patients in the entire set, and the results showed that the 10 lncRNAs could better classify patients into different risk patterns. (**Figures 3A–C**).

### ROC analysis and survival analysis based on the risk model

We separated patients of the two sets into high-risk and low-risk groups based on the median of the risk score in the training set to investigate the risk model’s prognostic value. In both groups, patients in the high-risk group had substantially lower overall survival (OS) and progression-free survival (PFS) than those in the low-risk group. (**Figures 3D, E**). The ROC curves confirmed the prognostic value of the risk model, showing that the AUCs for predicting survival were 0.758, 0.852, and 0.884 at 1, 3, and 5 years in the training group, and those in the testing set were 0.720, 0.745 and 0.720 (**Figure 3F**), respectively. Interestingly, in the two sets, we found that high-risk patients exhibited higher mortality than low-risk patients. (**Figures 3G, H**). The RNA expression levels of the 10 CRLs in the risk models in two sets were shown in heatmaps (**Figures 3I, J**). These results confirmed the value of our model in predicting the prognosis of GC patients.

### Prognostic value of the risk model

**Figure 4:**
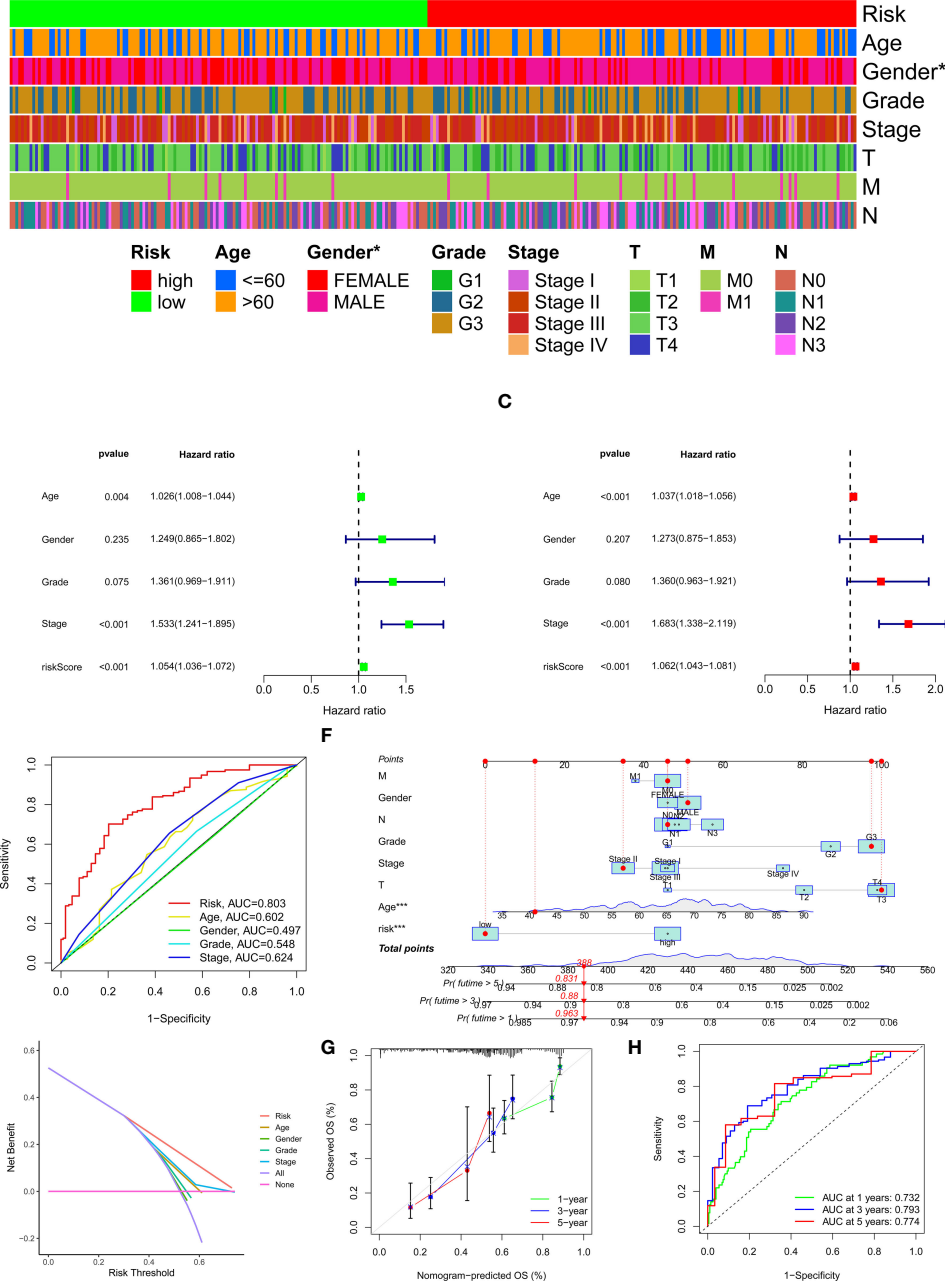
Clinical application of the risk model. **(A)** Correlation between the risk model and the clinicopathological characteristics of GC patients. **(B, C)** Univariate analysis and multivariate analysis were conducted to confirm the independent prognosis function of the model. **(D, E)** ROC, and DCA curves were performed to confirm the superiority of the risk score in clinical application. **(F)** The nomogram was plotted to obtain the predicting survival time of GC patients. **(G)** A calibration curve was applied to assess the accuracy of the model in predicting patients’ survival time. **(H)** The AUC value of the nomogram. *p < 0.05, and ***p < 0.001.

**Figure 5:**
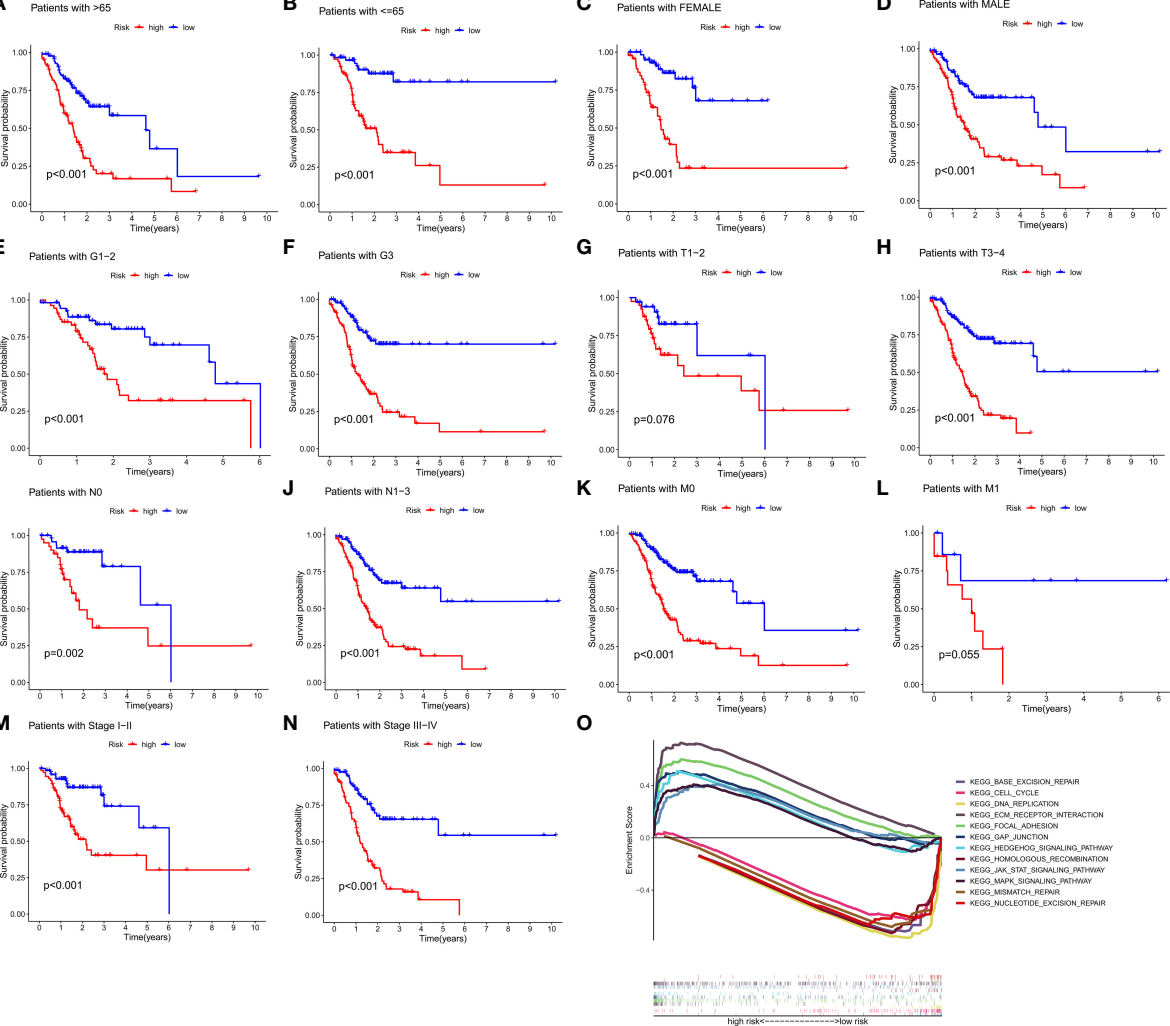
The prognostic function of the risk model and GSEA of 10 lncRNAs in the risk model. **(A–N)** The survival outcome of patients with different risk patterns was assessed in different subgroups. **(O)** GSEA results of 10 lncRNAs were visualized. (p < 0.05).

To investigate the relationship between clinical characteristics and the risk model in GC patients, we analyzed the overall distribution of different clinical characteristics between the two risk groups based on the 10 CRLs expression. Subsequently, we analyzed the correlation between the different risk groups and the corresponding clinical characteristics, and the results showed that elevated risk scores correlated with the gender of the patients (*p* < 0.05), but not with other clinical characteristics (**Figure 4A**). Next, to evaluate whether the risk score could be applied as an independent prognostic factor in predicting the prognosis of GC patients, we performed univariate and multivariate analyses, which confirmed that the risk score was a high-risk factor and could be applied as an independent prognostic factor in predicting survival in GC patients (**Figures 4B, C**). To further validate the value of the risk score as a predictor of prognosis over other clinical characteristics for GC patients, we plotted clinically relevant ROC curves and decision curves and found that the risk score was the most valuable predictor of prognosis compared to other clinical characteristics (**Figures 4D, E**). Besides, we plotted a nomogram to gain the predicted survival time for GC patients, and we established calibration curves and ROC curves to determine the accuracy of it (**Figures 4F–H**). To further confirm the prognostic value of the risk model among patients with different clinical features, we separated the patients into two different subgroups based on their clinical characteristics and assessed the survival differences of patients with different risk patterns within the different subgroups. We found that except for the M1 and T1-2 subgroups, where there was no statistical difference in survival of patients, patients with high risk showed a worse prognostic outcome than low-risk patients in all other subgroups (**Figures 5A–N**).

### Gene set enrichment analysis

Gene set enrichment analysis (GSEA) revealed that numerous pathways were activated in high-risk patients, and these pathways were mainly involved in Focal adhesion, ECM receptor interaction, Gap junction, Hedgehog signaling pathway, MAPK signaling pathway, and JAK-STAT signaling pathway, suggesting that these CRLs may be directly related to the development of gastric cancer. The pathways activated in low-risk patients mainly include Base excision repair, DNA replication, Cell cycle, Homologous recombination, Mismatch repair, and Nucleotide excision repair, and the activation of these pathways may somehow promote normal cell cycle progression and thus inhibit tumor development (**Figure 5O**).

### Comprehensive immune infiltration analysis based on risk signature subgroups

**Figure 6:**
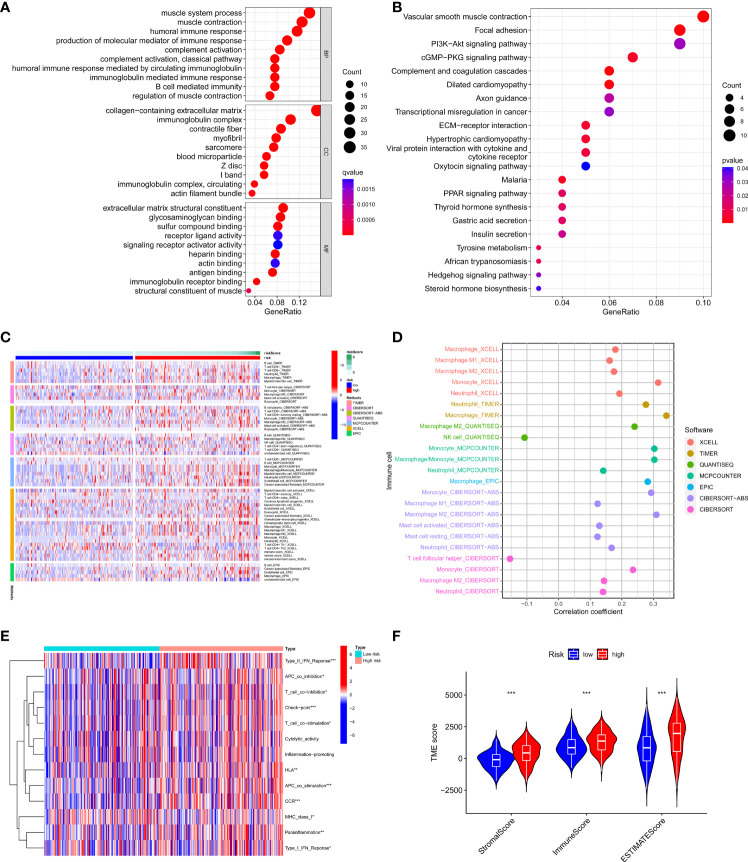
Relationship between the risk model and tumor immune microenvironment. **(A, B)** GO, and KEGG analysis of differentially expressed genes between high-risk and low-risk groups. **(C, D)** The infiltration status of immune cells of patients with different risk patterns. **(E)** Immune function differences of patients with different risk patterns. **(F)** The score of tumor immune microenvironment in different risk groups. *p < 0.05, **p < 0.01, and ***p < 0.001.

We further analyzed differentially expressed genes between two risk groups and then performed Gene Ontology (GO) enrichment analysis and Kyoto Encyclopaedia of Genes and Genomes (KEGG) pathway analysis based on these genes. The results showed that the genes were mainly associated with the immune response, cGMP−PKG signaling pathway, and PI3K−Akt signaling pathway (**Figures 6A, B**). Based on the functional analyses, we sought to investigate the relationship between risk models and the tumor immune microenvironment. Then we downloaded TCGA tumor immune infiltration data from TIMER 2.0 and visualized the results of immune cell infiltration differences between high-risk and low-risk patients *via* heatmap based on six algorithms (QUANTISEQ, MCPCOUNTER, XCELL, EPIC, CIBERSORT, and TIMER) (**Figure 6C**). Interestingly, we observed the infiltration of helper T cells and NK cells was negatively associated with the risk score, while the relationship between the risk score and the infiltration of macrophages, mast cells, neutrophils and monocytes was just the opposite (**Figure 6D**). Immune function analysis revealed significant differences in immune function between the two risk groups except for cytolytic activity, inflammation-promoting, and the relevant immune functions were more active in the high-risk group (**Figure 6E**). In addition, we scored the tumor microenvironment for each sample in the dataset, including immune score, stromal score, and estimate score, and then we analyzed the differences in these scores between the two risk groups. The results showed that the high-risk group had higher levels of immune infiltration score, stromal score, and a higher estimate score compared to the low-risk group (**Figure 6F**). These results suggest that our risk model can be applied to predict the immune profile of patients with GC.

### Clinical application of the risk model

**Figure 7:**
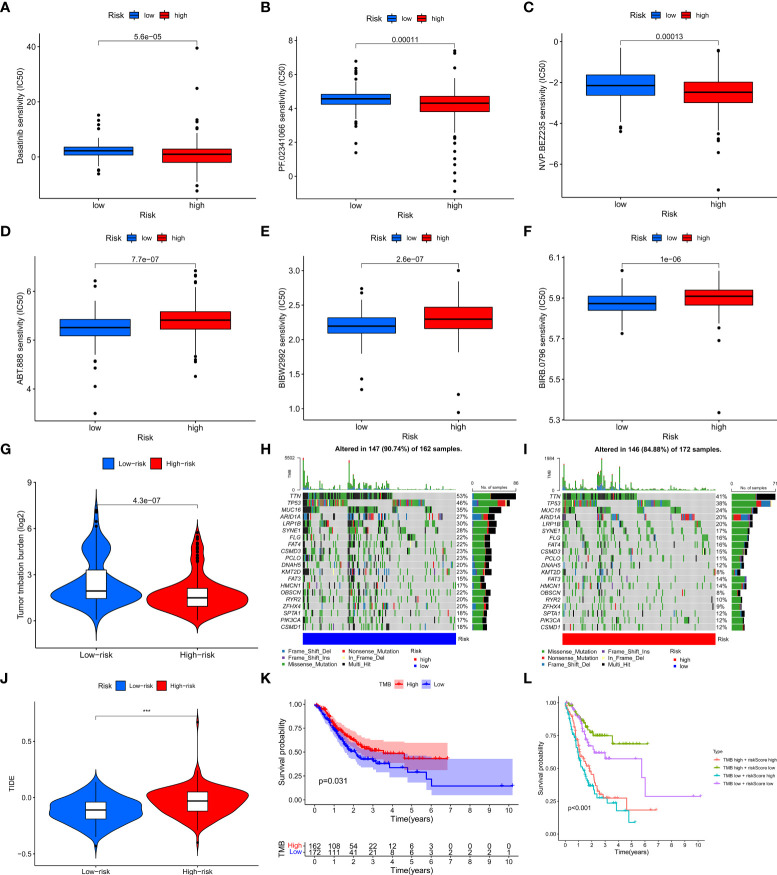
The relationship between the risk model and immunotherapy or TMB. **(A–F)** Drug sensitivity of patients with different risk patterns. **(G)** The relationship between risk score value and TMB level. **(H, I)** TMB status of the top 20 genes was visualized in two groups. **(J)** TIDE score of patients in two risk groups. **(K)** The survival outcome of the patients with different TMB levels. **(L)** The prognosis of patients with different tumor mutation burdens and different risk patterns was accessed. ***p < 0.001.

In order to evaluate the value of the clinical application of the risk model, we examined the difference in sensitivity of chemotherapeutic agents/targeted drugs between two risk groups. The results revealed that high-risk patients were more sensitive to dasatinib, crizotinib (PF.02341066), Dactolisib (NVP.BEZ235), etc., while low-risk patients were more sensitive to Afatinib (BIBW2992), ABT.888 (PARP inhibitor), BIRB.0796 (P38 MAPK inhibitor) (**Figures 7A–F**).

### Relationship between tumor mutation burden and the risk model

To investigate whether there is a correlation between tumor mutation burden (TMB) and the risk model, we acquired TMB data of GC patients from TCGA and compared the difference in TMB between the two risk groups. The results showed that the high-risk group had a lower level of TMB and the risk score was negatively associated with TMB levels (**Figures 7G**, **S1K**). In addition, we visualized the top 20 mutated genes between the two risk groups and observed that these 20 genes had significantly lower mutation levels in the high-risk group than in the low-risk group (**Figures 7H, I**). As to the relationship between TMB and immunotherapy, a tumor immune dysfunction and exclusion (TIDE) analysis was conducted to evaluate whether there are differences in immunotherapy responses among patients with different risk patterns. The results showed that the low-risk group had a lower TIDE score, indicating a better response to immunotherapy (**Figure 7J**). We then assessed the prognosis of patients with different TMB levels and different risk patterns and observed that low-risk patients with high TMB had the best prognosis, while high-risk patients with low TMB had the worst prognosis (**Figures 7K, L**)

### Expression of CRLs in GC samples

**Figure 8:**
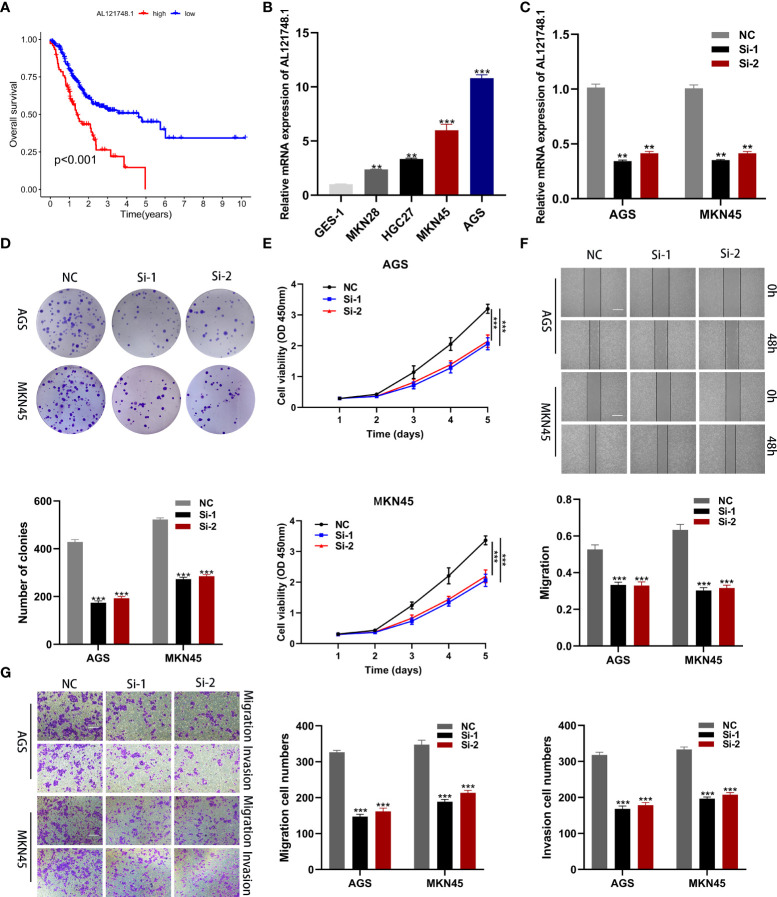
Cellular functional experiment. **(A)** Survival analysis of AL121748.1 in GC. **(B)** qRT-PCR to evaluate the expression of AL121748.1 in GC cell lines and GES-1. **(C)** qRT-PCR to evaluate the transfection efficiency. **(D)** Colony formation assay was conducted to detect the proliferation ability of GC cells after transfection. **(E)** The effect of AL121748.1 on proliferation was explored by the CCK-8 assay. **(F)** Wound healing assay was used to determine the effect of migration of AL121748.1(scale bar = 100 μm). **(G)** Transwell assays were used to show the effect of AL121748.1 on GC cell migration and invasion (scale bar = 200 μm). **p < 0.01, and ***p < 0.001.

To identify the key genes in the risk model, we examined the expression differences of 10 CRLs in normal and tumor tissues, and the results revealed that all 10 lncRNAs in the risk model were highly expressed in tumor tissues (**Figures S1A-J**). Interestingly, we found that AL121748.1 exhibited the highest HR value among these 10 lncRNAs (**Figure 2E**), and we subsequently conducted a survival analysis on AL121748.1. We found that patients with a high expression level of AL121748.1 exhibited a poorer prognosis than those with a low expression level (**Figure 8A**).

### AL121748.1 is upregulated in GC cells and promotes GC cell vitality and proliferation

We explored AL121748.1 expression in GC cell lines and found that it was highly expressed in the GC cell lines, especially in MKN45 and AGS (**Figure 8B**). Thus, we knocked down AL121748.1 expression in MKN45 and AGS with siRNA (**Figure 8C**). Then we conducted CCK-8 and colony formation assay in the two cell lines. The results revealed that cells with a reduced expression of AL121748.1 exhibited a lower vitality and a substantial drop in the number of colonies when compared with the siRNA negative control (**Figures 8D, E**). These results suggested that AL121748.1 might play a crucial role in GC cell survival and proliferation.

### AL121748.1 promotes migration and invasion of GC cells

The wound healing assay showed that a significant reduction in wound healing rate was detected in cells with decreased AL121748.1 gene expression (**Figure 8F**). We further evaluated the migration and invasion capacity of GC cells after knocking down AL121748.1 *via* transwell assay. The results revealed that the AL121748.1 knockdown reduced both GC cell migration and invasion. (**Figure 8G**). These results confirmed that AL121748.1 could promote migration and invasion of GC cells.

## Discussion

Copper (Cu), an essential mineral nutrient for all organisms, is involved in a variety of biological processes such as mitochondrial respiration, antioxidant/detoxification processes, and iron uptake (19). For its redox properties, it is both beneficial and toxic to cells (17). Accumulated evidence suggests that Cu is not limited to being a static cofactor, but that it is also a dynamic signaling element with considerable effects on a variety of processes including lipolysis, autophagy, cytotoxicity, cell proliferation, and oxidative stress (18, 20, 21). Cellular copper homeostasis is essential for cell maintenance and metabolism, and disorders of copper metabolism are connected with the development of many diseases (22–24). As to the relationship between copper and cancer, numerous observations suggest that tumors need higher concentrations of copper compared to normal tissue (25). According to previous research, copper concentrations were elevated in tumors or serum from animal models and patients with a variety of cancers, including breast (26–28), lung (29), gastrointestinal (30–32), oral (33), thyroid (34), gallbladder (35), gynecological (36), and prostate cancers (37). In humans, mutations that cause excessive copper accumulation are life-threatening, but there may be a window in which a more concentrated increase in intracellular copper can be used to kill cancer cells selectively (17, 38). According to the research conducted by Tsvetkov and colleagues, copper toxicity involves the disruption of specific mitochondrial metabolic enzymes, which triggers an unusual cell death mechanism defined as cuproptosis, mediated by an ancient mechanism: protein lipoylation, which differs from all other known regulated cell death mechanisms such as apoptosis, pyroptosis, ferroptosis and necroptosis (16). This mechanism could explain the pathology associated with hereditary copper overload diseases and help to harness copper toxicity for cancer treatment (16).

In this study, we first identified 19 CRGs from previous research and we found that most of them were differentially expressed in GC. To further explored the CRLs, we conducted Pearson’s correlation analysis and achieved 1218 lncRNAs for the following analysis. Based on this, we obtained 27 prognostic lncRNAs *via* univariate COX analysis. We then established a 10 lncRNAs-based risk model *via* LASSO Cox regression analysis and multivariate COX analysis. As to the 10 CRLs in the risk model, AL121748.1 (39), LINC01980 (40), AL355574.1 (41), AL391152.1 (42), TYMSOS (43, 44), AC016737.1, AL512506.1 (45), AC104809.2 (46) were reported to have a relationship with various tumors, however, other lncRNAs were first reported. Due to cuproptosis being a novel discovered form of regulating cell death, there are few direct studies about it. To our knowledge, the 10 lncRNAs did not show any relationship with cuproptosis according to the previous research. Although we have found no research evidence that the 10 lncRNAs are directly related to cuproptosis, our research may supply significant clues to exploring the relationship between these lncRNAs and cuproptosis.

Then, we assigned all GC patients into two risk groups, and PCA results revealed that the model could classify patients into a high-risk or low-risk group efficiently. In addition, patients with high risk exhibited worse survival outcomes than those with low risk. To determine the accuracy of the model, we conducted ROC curves, and the AUCs values exceed 0.75 at one, three, and five years, meanwhile, the maximum AUC value (0.884) was detected at 5 years. We then confirmed the model we established could be assumed as an independent prognostic indicator *via* univariate and multivariate analyzes. Besides, the clinically relevant ROC curve and decision curve revealed that the risk score outperforms other clinical characteristics in terms of clinical application efficiency. Moreover, we also found that patients with low risk exhibit a better survival outcome than those with high risk in most subgroups except patients with T1-2 and M1. These results indicated that the model could be applied to predict the prognosis of GC patients.

The tumor immune microenvironment plays a significant role in tumor development. Differential infiltration of various immune cells in tumors might have an influence on patients’ prognosis (47, 48). In our study, we explored the level of immune cell infiltration, immunotherapy score, and TMB in GC patients with different risk patterns. High infiltration levels of NK cells and helper T cells are correlated with better prognosis, while the same infiltration levels of macrophages, mast cells, neutrophils, and monocytes are correlated with poor tumor prognosis (49–55). Besides, macrophages, mast cells, neutrophils, and monocytes have been reported to promote tumor progression (52, 56–59). In our study, we observed the infiltration of NK cells and T cells was negatively correlated with the risk score, while the correlation between the risk score and the infiltration of macrophages, mast cells, neutrophils, and monocytes was just the opposite. The results suggested that patients with high risk may show worse survival outcomes, which was consistent with the prognosis we analyzed. Thus, our risk model performs well in predicting immune infiltration in patients with GC.

As to TMB, previous research suggested that patients with a higher level of TMB may show a better sensitivity to immunotherapy (60). In our study, we found that the TMB level was inversely related to the risk score, which suggested that the low TMB level might perform as a protective factor. Thus, we performed survival analysis and found that low-risk patients with a high tumor mutation burden had the best prognosis, whereas high-risk patients with a low tumor mutation burden had the worst prognosis. According to previous research, we found that a variety of factors may affect the efficacy of immune checkpoint inhibition therapy, including mutation or neoantigen load, the level of cytotoxic T cell infiltration, PD-L1 levels, defective antigen presentation, defective mismatch repair, interferon signaling, tumor aneuploidy, and gut microbes. While the TIDE score combines T cell dysfunction and exclusion characteristics to simulate tumor immune escape with varying levels of tumor-infiltrating cytotoxic T cells and is superior to other biomarkers used to predict the efficacy of immune checkpoint inhibition therapy (61). Based on this, we evaluated the TIDE scores of the two groups and we found that the high-risk group has a higher TIDE score, which suggested patients in this group may be less sensitive to immunotherapy. These findings offered additional evidence that our risk model is related to the immunological landscape and can be applied to predict the prognosis of GC patients

AL121748.1 has been identified as a ferroptosis-related lncRNA that is related to immunotherapy and chemotherapy responses in GC patients. However, there is no experimental evidence for its role in GC. AL121748.1 was first identified as the most significant HR among the ten modeled lncRNAs in our research, and further survival analysis revealed that high expression of AL121748.1 was related to a bad prognosis in GC patients. Cellular functional experiments proved that knocking down AL121748.1 in GC cell lines greatly reduced GC cell activity, proliferation, migration, and invasion. This suggests a possible treatment target for GC.

## Conclusion

In conclusion, we established a 10 CRLs-based risk model. The model performed better in predicting the prognosis of GC patients than other clinical characteristics. Besides, the model was significantly related to tumor immune microenvironment, tumor mutation burden, drug sensitivity, and immunotherapy response of GC patients. Furthermore, we confirmed the oncogenic role of AL121748.1 in GC *via* cellular functional experiments. These findings provided critical clues for future immunological research on GC as well as a potential therapeutic target for GC.

## Data availability statement

The original contributions presented in the study are included in the article/**Supplementary Material**. Further inquiries can be directed to the corresponding author.

## Author contributions

YW for study concept and design, acquisition of data, analysis, and interpretation of data, statistical analysis, and drafting of the paper. KS, KL, XZ, QC, LH for the acquisition of data, analysis, and interpretation of data, and statistical analysis. HF, PN for technical and material support. JX for analysis and interpretation of data, critical revision of the paper. ZX for study supervision. DZ for statistical analysis, critical revision of the paper for important intellectual content, study supervision. LY for obtaining funding, critical revision of the paper for important intellectual content, and study supervision. All authors contributed to the article and approved the submitted version.

## Funding

This study was financially supported by the National Natural Science Foundation of China (Grant no. 81874219).

## Conflict of interest

The authors declare that the research was conducted in the absence of any commercial or financial relationships that could be construed as a potential conflict of interest.

## Publisher’s note

All claims expressed in this article are solely those of the authors and do not necessarily represent those of their affiliated organizations, or those of the publisher, the editors and the reviewers. Any product that may be evaluated in this article, or claim that may be made by its manufacturer, is not guaranteed or endorsed by the publisher.

## Abbreviations

GC, gastric cancer; TCGA, The Cancer Genome Atlas; AGC, advanced gastric cancer; MSI, microsatellite instability; LncRNA, Long non-coding RNA; CRGs, cuproptosis-related genes; CNV, copy number variation; CRLs, cuproptosis-related lncRNAs; TCA, tricarboxylic acid; PCA, principal components analysis; IC50, 50% inhibiting concentration (IC50); TMB, Tumor mutation burden; OS, overall survival; PFS, progression-free survival; GSEA, Gene Set Enrichment Analysis; GO, Gene Ontology; KEGG, Kyoto Encyclopaedia of Genes and Genomes; TIDE, Tumor Immune Dysfunction and Exclusion.

## References

[1] Sung H Ferlay J Siegel RL Laversanne M Soerjomataram I Jemal A. Global cancer statistics 2020: Globocan estimates of incidence and mortality worldwide for 36 cancers in 185 countries. CA: Cancer J For Clin (2021) 71(3):209–49. doi: 10.3322/caac.21660 33538338

[2] Nakamura Y Kawazoe A Lordick F Janjigian YY Shitara K. Biomarker-targeted therapies for advanced-stage gastric and gastro-oesophageal junction cancers: An emerging paradigm. Nat Rev Clin Oncol (2021) 18(8):473–87. doi: 10.1038/s41571-021-00492-2 33790428

[3] Kopp F Mendell JT. Functional classification and experimental dissection of long noncoding rnas. Cell (2018) 172(3):393–407. doi: 10.1016/j.cell.2018.01.011 29373828 PMC5978744

[4] Lin C Yang L. Long noncoding rna in cancer: Wiring signaling circuitry. Trends Cell Biol (2018) 28(4):287–301. doi: 10.1016/j.tcb.2017.11.008 29274663 PMC5869122

[5] Khorkova O Hsiao J Wahlestedt C. Basic biology and therapeutic implications of lncrna. Adv Drug Delivery Rev (2015) 87:15–24. doi: 10.1016/j.addr.2015.05.012 PMC4544752 26024979

[6] Tan H Zhang S Zhang J Zhu L Chen Y Yang H. Long non-coding rnas in gastric cancer: New emerging biological functions and therapeutic implications. Theranostics (2020) 10(19):8880–902. doi: 10.7150/thno.47548 PMC7392009 32754285

[7] Xie S Chang Y Jin H Yang F Xu Y Yan X. Non-coding rnas in gastric cancer. Cancer Lett (2020) 493:55–70. doi: 10.1016/j.canlet.2020.06.022 32712234

[8] Li Y Wu Z Yuan J Sun L Lin L Huang N. Long non-coding rna Malat1 promotes gastric cancer tumorigenicity and metastasis by regulating vasculogenic mimicry and angiogenesis. Cancer Lett (2017) 395:31–44. doi: 10.1016/j.canlet.2017.02.035 28268166

[9] Sun L Li J Yan W Yao Z Wang R Zhou X. H19 promotes aerobic glycolysis, proliferation, and immune escape of gastric cancer cells through the microrna-519d-3p/Lactate dehydrogenase a axis. Cancer Sci (2021) 112(6):2245–59. doi: 10.1111/cas.14896 PMC8177792 33756038

[10] Sun M Nie F Wang Y Zhang Z Hou J He D. Lncrna Hoxa11-as promotes proliferation and invasion of gastric cancer by scaffolding the chromatin modification factors Prc2, Lsd1, and Dnmt1. Cancer Res (2016) 76(21):6299–310. doi: 10.1158/0008-5472.CAN-16-0356 27651312

[11] Zhao J Du P Cui P Qin Y Ce Hu Wu J. Lncrna Pvt1 promotes angiogenesis *Via* activating the Stat3/Vegfa axis in gastric cancer. Oncogene (2018) 37(30):4094–109. doi: 10.1038/s41388-018-0250-z 29706652

[12] Dan J Wang J Wang Y Zhu M Yang X Peng Z. Lncrna-Meg3 inhibits proliferation and metastasis by regulating mirna-21 in gastric cancer. BioMed Pharmacother (2018) 99:931–8. doi: 10.1016/j.biopha.2018.01.164 29710493

[13] Peng W Si S Zhang Q Li C Zhao F Wang F. Long non-coding rna Meg3 functions as a competing endogenous rna to regulate gastric cancer progression. J Exp Clin Cancer Res (2015) 34:79. doi: 10.1186/s13046-015-0197-7 26253106 PMC4529701

[14] Qi P M-d Xu Shen X-H Ni S-J Huang D Tan C. Reciprocal repression between Tusc7 and mir-23b in gastric cancer. Int J Cancer (2015) 137(6):1269–78. doi: 10.1002/ijc.29516 25765901

[15] Tang D Chen X Kroemer G. Cuproptosis: A copper-triggered modality of mitochondrial cell death. Cell Res (2022) 32(5):417–8. doi: 10.1038/s41422-022-00653-7 PMC9061796 35354936

[16] Tsvetkov P Coy S Petrova B Dreishpoon M Verma A Abdusamad M. Copper induces cell death by targeting lipoylated tca cycle proteins. Science (2022) 375(6586):1254–61. doi: 10.1126/science.abf0529 PMC9273333 35298263

[17] Ge EJ Bush AI Casini A Cobine PA Cross JR DeNicola GM. Connecting copper and cancer: From transition metal signalling to metalloplasia. Nat Rev Cancer (2022) 22(2):102–13. doi: 10.1038/s41568-021-00417-2 PMC8810673 34764459

[18] Cobine PA Moore SA Leary SC. Getting out what you put in: Copper in mitochondria and its impacts on human disease. Biochim Biophys Acta Mol Cell Res (2021) 1868(1):118867. doi: 10.1016/j.bbamcr.2020.118867 32979421 PMC7680424

[19] Ruiz LM Libedinsky A Elorza AA. Role of copper on mitochondrial function and metabolism. Front Mol Biosci (2021) 8:711227. doi: 10.3389/fmolb.2021.711227 34504870 PMC8421569

[20] Hu Y Qian Y Wei J Jin T Kong X Cao H. The Disulfiram/Copper complex induces autophagic cell death in colorectal cancer by targeting Ulk1. Front Pharmacol (2021) 12:752825. doi: 10.3389/fphar.2021.752825 34887757 PMC8650091

[21] Tsang T Posimo JM Gudiel AA Cicchini M Feldser DM Brady DC. Copper is an essential regulator of the autophagic kinases Ulk1/2 to drive lung adenocarcinoma. Nat Cell Biol (2020) 22(4):412–24. doi: 10.1038/s41556-020-0481-4 PMC7610258 32203415

[22] Bandmann O Weiss KH Kaler SG. Wilson's disease and other neurological copper disorders. Lancet Neurol (2015) 14(1):103–13. doi: 10.1016/S1474-4422(14)70190-5 PMC4336199 25496901

[23] Członkowska A Litwin T Dusek P Ferenci P Lutsenko S Medici V. Wilson Disease. Nat Rev Dis Primers (2018) 4(1):21. doi: 10.1038/s41572-018-0018-3 30190489 PMC6416051

[24] Kaler SG. Atp7a-related copper transport diseases-emerging concepts and future trends. Nat Rev Neurol (2011) 7(1):15–29. doi: 10.1038/nrneurol.2010.180 21221114 PMC4214867

[25] Blockhuys S Celauro E Hildesjö C Feizi A Stål O Fierro-González JC. Defining the human copper proteome and analysis of its expression variation in cancers. Metallomics (2017) 9(2):112–23. doi: 10.1039/c6mt00202a 27942658

[26] Ding X Jiang M Jing H Sheng W Wang X Han J. Analysis of serum levels of 15 trace elements in breast cancer patients in Shandong, China. Environ Sci pollut Res Int (2015) 22(10):7930–5. doi: 10.1007/s11356-014-3970-9 25520207

[27] Adeoti ML Oguntola AS Akanni EO Agodirin OS Oyeyemi GM. Trace elements; copper, zinc and selenium, in breast cancer afflicted female patients in lautech osogbo, Nigeria. Indian J Cancer (2015) 52(1):106–9. doi: 10.4103/0019-509X.175573 26837992

[28] Feng J-F Lu L Zeng P Yang Y-H Luo J Yang Y-W. Serum total Oxidant/Antioxidant status and trace element levels in breast cancer patients. Int J Clin Oncol (2012) 17(6):575–83. doi: 10.1007/s10147-011-0327-y 21968912

[29] Jin Y Zhang C Xu H Xue S Wang Y Hou Y. Combined effects of serum trace metals and polymorphisms of Cyp1a1 or Gstm1 on non-small cell lung cancer: A hospital based case-control study in China. Cancer Epidemiol (2011) 35(2):182–7. doi: 10.1016/j.canep.2010.06.004 20638923

[30] Stepien M Jenab M Freisling H Becker N-P Czuban M Tjønneland A. Pre-diagnostic copper and zinc biomarkers and colorectal cancer risk in the European prospective investigation into cancer and nutrition cohort. Carcinogenesis (2017) 38(7):699–707. doi: 10.1093/carcin/bgx051 28575311

[31] Sohrabi M Gholami A Azar MH Yaghoobi M Shahi MM Shirmardi S. Trace element and heavy metal levels in colorectal cancer: Comparison between cancerous and non-cancerous tissues. Biol Trace Elem Res (2018) 183(1):1–8. doi: 10.1007/s12011-017-1099-7 28795369

[32] Ribeiro S Moya AMTM Braga CBM Domenici FA Feitosa MR Feres O. Copper-zinc ratio and nutritional status in colorectal cancer patients during the perioperative period. Acta Cir Bras (2016) 31 Suppl 1:24–8. doi: 10.1590/S0102-86502016001300006 27142901

[33] Khanna SS Karjodkar FR. Circulating immune complexes and trace elements (Copper, iron and selenium) as markers in oral precancer and cancer : A randomised, controlled clinical trial. Head Face Med (2006) 2:33. doi: 10.1186/1746-160X-2-33 17040577 PMC1629009

[34] Baltaci AK Dundar TK Aksoy F Mogulkoc R. Changes in the serum levels of trace elements before and after the operation in thyroid cancer patients. Biol Trace Elem Res (2017) 175(1):57–64. doi: 10.1007/s12011-016-0768-2 27263537

[35] Basu S Singh MK Singh TB Bhartiya SK Singh SP Shukla VK. Heavy and trace metals in carcinoma of the gallbladder. World J Surg (2013) 37(11):2641–6. doi: 10.1007/s00268-013-2164-9 23942528

[36] Yaman M Kaya G Yekeler H. Distribution of trace metal concentrations in paired cancerous and non-cancerous human stomach tissues. World J Gastroenterol (2007) 13(4):612–8. doi: 10.3748/wjg.v13.i4.612 PMC4065986 17278230

[37] Saleh SAK Adly HM Abdelkhaliq AA Nassir AM. Serum levels of selenium, zinc, copper, manganese, and iron in prostate cancer patients. Curr Urol (2020) 14(1):44–9. doi: 10.1159/000499261 PMC7206590 32398996

[38] Kahlson MA Dixon SJ. Copper-induced cell death. Science (2022) 375(6586):1231–2. doi: 10.1126/science.abo3959 35298241

[39] Lai D Tan L Zuo X Liu D Jiao D Wan G. Prognostic ferroptosis-related lncrna signatures associated with immunotherapy and chemotherapy responses in patients with stomach cancer. Front Genet (2021) 12:798612. doi: 10.3389/fgene.2021.798612 35047016 PMC8762254

[40] Liang X Wu Z Shen S Niu Y Guo Y Liang J. Linc01980 facilitates esophageal squamous cell carcinoma progression *Via* regulation of mir-190a-5p/Myo5a pathway. Arch Biochem Biophys (2020) 686:108371. doi: 10.1016/j.abb.2020.108371 32325088

[41] Chen D Wang M Xu Y Jiang X Xiong L Zhang L. A novel autophagy-related lncrna prognostic signature associated with immune microenvironment and survival outcomes of gastric cancer patients. Int J Gen Med (2021) 14:6935–50. doi: 10.2147/IJGM.S331959 PMC8541751 34703297

[42] Liu M Li J Huang Z Li Y. Gastric cancer risk-scoring system based on analysis of a competing endogenous rna network. Transl Cancer Res (2020) 9(6):3889–902. doi: 10.21037/tcr-19-2977 PMC8798172 35117756

[43] Gu Y Wan C Zhou G Zhu J Shi Z Zhuang Z. Tymsos drives the proliferation, migration, and invasion of gastric cancer cells by regulating Znf703 *Via* sponging mir-4739. Cell Biol Int (2021) 45(8):1710–9. doi: 10.1002/cbin.11610 33847425

[44] Yuan Y Jiang X Tang L Wang J Zhang D Cho WC. Foxm1/Lncrna Tymsos/Mir-214-3p-Mediated high expression of ncapg correlates with poor prognosis and cell proliferation in non-small cell lung carcinoma. Front Mol Biosci (2021) 8:785767. doi: 10.3389/fmolb.2021.785767 35211508 PMC8862726

[45] Zhang S Li X Tang C Kuang W. Inflammation-related long non-coding rna signature predicts the prognosis of gastric carcinoma. Front Genet (2021) 12:736766. doi: 10.3389/fgene.2021.736766 34819945 PMC8607501

[46] Cao Q Dong Z Liu S An G Yan B Lei L. Construction of a metastasis-associated cerna network reveals a prognostic signature in lung cancer. Cancer Cell Int (2020) 20:208. doi: 10.1186/s12935-020-01295-8 32518519 PMC7271455

[47] Xu C Sui S Shang Y Yu Z Han J Zhang G. The landscape of immune cell infiltration and its clinical implications of pancreatic ductal adenocarcinoma. J Adv Res (2020) 24:139–48. doi: 10.1016/j.jare.2020.03.009 PMC7171261 32322419

[48] Edlund K Madjar K Mattsson JSM Djureinovic D Lindskog C Brunnström H. Prognostic impact of tumor cell programmed death ligand 1 expression and immune cell infiltration in nsclc. J Thorac Oncol (2019) 14(4):628–40. doi: 10.1016/j.jtho.2018.12.022 30639618

[49] Ugel S Canè S De Sanctis F Bronte V. Monocytes in the tumor microenvironment. Annu Rev Pathol (2021) 16:93–122. doi: 10.1146/annurev-pathmechdis-012418-013058 33497262

[50] Gao M Lin Y Liu X Zhao Z Zhu Z Zhang H. Mutation is accompanied by neutrophil infiltration and contributes to poor survival in isocitrate dehydrogenase wild-type glioma. Front Cell Dev Biol (2021) 9:654407. doi: 10.3389/fcell.2021.654407 33996815 PMC8119999

[51] Hu G Wang S Cheng P. Tumor-infiltrating tryptase mast cells predict unfavorable clinical outcome in solid tumors. Int J Cancer (2018) 142(4):813–21. doi: 10.1002/ijc.31099 29023696

[52] Cassetta L Pollard JW. Targeting macrophages: Therapeutic approaches in cancer. Nat Rev Drug Discovery (2018) 17(12):887–904. doi: 10.1038/nrd.2018.169 30361552

[53] Kim HS Kim MG Min K-W Jung US Kim D-H. High mmp-11 expression associated with low Cd8+ T cells decreases the survival rate in patients with breast cancer. PLoS One (2021) 16(5):e0252052. doi: 10.1371/journal.pone.0252052 34038440 PMC8153507

[54] Cursons J Souza-Fonseca-Guimaraes F Foroutan M Anderson A Hollande F Hediyeh-Zadeh S. A gene signature predicting natural killer cell infiltration and improved survival in melanoma patients. Cancer Immunol Res (2019) 7(7):1162–74. doi: 10.1158/2326-6066.CIR-18-0500 31088844

[55] Melssen M Slingluff CL. Vaccines targeting helper T cells for cancer immunotherapy. Curr Opin Immunol (2017) 47:85–92. doi: 10.1016/j.coi.2017.07.004 28755541 PMC5757837

[56] Granot Z. Neutrophils as a therapeutic target in cancer. Front Immunol (2019) 10:1710. doi: 10.3389/fimmu.2019.01710 31379884 PMC6659000

[57] Sammarco G Varricchi G Ferraro V Ammendola M De Fazio M Altomare DF. Mast cells, angiogenesis and lymphangiogenesis in human gastric cancer. Int J Mol Sci (2019) 20(9). doi: 10.3390/ijms20092106 PMC6540185 31035644

[58] Lv Y Zhao Y Wang X Chen N Mao F Teng Y. Increased intratumoral mast cells foster immune suppression and gastric cancer progression through tnf-A-Pd-L1 pathway. J Immunother Cancer (2019) 7(1):54. doi: 10.1186/s40425-019-0530-3 30808413 PMC6390584

[59] Peng L-S Zhang J-Y Teng Y-S Zhao Y-L Wang T-T Mao F-Y. Tumor-associated Monocytes/Macrophages impair nk-cell function *Via* Tgfβ1 in human gastric cancer. Cancer Immunol Res (2017) 5(3):248–56. doi: 10.1158/2326-6066.CIR-16-0152 28148545

[60] Allgäuer M Budczies J Christopoulos P Endris V Lier A Rempel E. Implementing tumor mutational burden (Tmb) analysis in routine diagnostics-a primer for molecular pathologists and clinicians. Transl Lung Cancer Res (2018) 7(6):703–15. doi: 10.21037/tlcr.2018.08.14 PMC6249620 30505715

[61] Jiang P Gu S Pan D Fu J Sahu A Hu X. Signatures of T cell dysfunction and exclusion predict cancer immunotherapy response. Nat Med (2018) 24(10):1550–8. doi: 10.1038/s41591-018-0136-1 PMC6487502 30127393

