# Supplementary material for: A novel risk model construction and immune landscape analysis of gastric cancer based on cuproptosis-related long noncoding RNAs
Source: Front Oncol. 2022 Oct 26;12:1015235. doi: 10.3389/fonc.2022.1015235 (PMC9643840; doi:10.3389/fonc.2022.1015235)
Supplement: Supplementary file 2 [file Presentation_1.zip › Supplementary Materials and Methods.docx]

**Supplementary Materials and Methods**

*Cell lines and cell transfection experiments*

The four human GC cell lines used in this study, AGS, MKN45, HGC-27, MKN-28, and normal human gastric epithelial cell line GES-1, were obtained from the Chinese Academy of Sciences' cell bank (Shanghai, China). All cell lines were cultured in RPMI 1640 medium (Invitrogen) containing 10% fetal bovine serum (FBS, WISENT, Canada) and 1% antibiotics (100 U/ml penicillin and 100 mg/ml streptomycin) at 37 °C and 5% CO2. Lipofectamine 3000 (Invitrogen) was used to transfect small interfering RNA (siRNA) into GC cells for transfection studies. The siRNA sequences designed against AL121748.1 are listed in Supplementary Table 3.

*RNA extraction and quantitative real‑time PCR*

Total RNA was extracted with TRIzol reagent (Invitrogen, Carlsbad, CA, USA) according to the manufacturer's instructions and was reverse-transcribed to cDNA with a Prime Script RT reagent Kit (Takara, China). Then cDNA was amplified with a 7500 Real-Time PCR System (Applied Biosystems, USA) with Universal SYBR Green Master Mix (Roche, Shanghai, China). The results were normalized to GAPDH expression. The information of primers used was shown in Supplementary Table 4. All procedures were performed in triplicate and the 2^−ΔΔCT^ method was applied to calculate relative expression levels.

*Cell proliferation and colony formation assays*

Each well was inoculated with 1000 cells and the cell proliferation rate was detected with Cell Counting Kit 8 (CCK-8) (Djingo, Japan) for 5 d. The absorbance at 450 nm was recorded with a standard microplate reader (Scienific MultiskanMK3, Thermo Scientific). As to colony formation, cells in 6-well plates were incubated for two weeks at 37°C in a 5% CO2 incubator. then cells were stained, imaged and colony counts were performed.

*Wound healing and transwell assays*

Cells were inoculated in 6-well plates and cultured until fused. A linear scratch wound was formed with a 2 0 0 μl sterile pipette tip and rinsed with phosphate-buffered saline. Images were taken at 0 h and 48 h, respectively, and the cell healing rate was calculated based on cell coverage. As to transwell assay, transwell chambers with a membrane pore size of 8 μm were uncoated or coated with Matrigel (BD Biosciences, USA). 2×10^4^ cells were inoculated into a serum-free culture medium in the upper chamber and 10% fetal bovine serum in the lower chamber. After 24 h incubation, cells were fixed, stained, and counted by inverted microscopy.
