# Supplementary material for: A novel risk model construction and immune landscape analysis of gastric cancer based on cuproptosis-related long noncoding RNAs
Source: Front Oncol. 2022 Oct 26;12:1015235. doi: 10.3389/fonc.2022.1015235 (PMC9643840; doi:10.3389/fonc.2022.1015235)
Supplement: Supplementary file 3 [file Table_1.docx]

**Supplementary Table1** clinical characteristics of patients

| Covariates | Type | Total | Test | Train | Pvalue |
| --- | --- | --- | --- | --- | --- |
| Age | <=65 | 153(44.74%) | 73(42.94%) | 80(46.51%) | 0.6828 |
|  | >65 | 186(54.39%) | 94(55.29%) | 92(53.49%) |  |
|  | unknow | 3(0.88%) | 3(1.76%) | 0(0%) |  |
| Gender | FEMALE | 121(35.38%) | 57(33.53%) | 64(37.21%) | 0.5495 |
|  | MALE | 221(64.62%) | 113(66.47%) | 108(62.79%) |  |
| Grade | G1 | 9(2.63%) | 2(1.18%) | 7(4.07%) | 0.1995 |
|  | G2 | 121(35.38%) | 64(37.65%) | 57(33.14%) |  |
|  | G3 | 203(59.36%) | 100(58.82%) | 103(59.88%) |  |
|  | unknow | 9(2.63%) | 4(2.35%) | 5(2.91%) |  |
| Stage | Stage I | 45(13.16%) | 23(13.53%) | 22(12.79%) | 0.3001 |
|  | Stage II | 107(31.29%) | 55(32.35%) | 52(30.23%) |  |
|  | Stage III | 140(40.94%) | 62(36.47%) | 78(45.35%) |  |
|  | Stage IV | 36(10.53%) | 22(12.94%) | 14(8.14%) |  |
|  | unknow | 14(4.09%) | 8(4.71%) | 6(3.49%) |  |
| T | T1 | 15(4.39%) | 8(4.71%) | 7(4.07%) | 0.5532 |
|  | T2 | 74(21.64%) | 42(24.71%) | 32(18.6%) |  |
|  | T3 | 159(46.49%) | 75(44.12%) | 84(48.84%) |  |
|  | T4 | 90(26.32%) | 43(25.29%) | 47(27.33%) |  |
|  | unknow | 4(1.17%) | 2(1.18%) | 2(1.16%) |  |
| M | M0 | 305(89.18%) | 149(87.65%) | 156(90.7%) | 0.2822 |
|  | M1 | 24(7.02%) | 15(8.82%) | 9(5.23%) |  |
|  | unknow | 13(3.8%) | 6(3.53%) | 7(4.07%) |  |
| N | N0 | 99(28.95%) | 51(30%) | 48(27.91%) | 0.9794 |
|  | N1 | 92(26.9%) | 45(26.47%) | 47(27.33%) |  |
|  | N2 | 70(20.47%) | 35(20.59%) | 35(20.35%) |  |
|  | N3 | 70(20.47%) | 34(20%) | 36(20.93%) |  |
|  | unknow | 11(3.22%) | 5(2.94%) | 6(3.49%) |  |
| Radiotherapy | NO | 144(42.11%) | 67(39.41%) | 77(44.77%) | 0.3004 |
|  | unknow | 156(45.61%) | 79(46.47%) | 77(44.77%) |  |
|  | YES | 42(12.28%) | 24(14.12%) | 18(10.47%) |  |
| Chemotherapy | C | 1(0.29%) | 0(0%) | 1(0.58%) | 0.4491 |
|  | CF | 1(0.29%) | 1(0.59%) | 0(0%) |  |
|  | DCF | 3(0.88%) | 3(1.76%) | 0(0%) |  |
|  | DCFL | 1(0.29%) | 1(0.59%) | 0(0%) |  |
|  | Didox | 1(0.29%) | 0(0%) | 1(0.58%) |  |
|  | DOF | 1(0.29%) | 0(0%) | 1(0.58%) |  |
|  | Doxifluridine | 5(1.46%) | 4(2.35%) | 1(0.58%) |  |
|  | E | 1(0.29%) | 0(0%) | 1(0.58%) |  |
|  | ECF | 14(4.09%) | 7(4.12%) | 7(4.07%) |  |
|  | ECX | 5(1.46%) | 1(0.59%) | 4(2.33%) |  |
|  | EF | 1(0.29%) | 0(0%) | 1(0.58%) |  |
|  | EFP | 1(0.29%) | 1(0.59%) | 0(0%) |  |
|  | ELF | 13(3.8%) | 3(1.76%) | 10(5.81%) |  |
|  | EOX | 7(2.05%) | 4(2.35%) | 3(1.74%) |  |
|  | EOXT | 1(0.29%) | 1(0.59%) | 0(0%) |  |
|  | F | 40(11.7%) | 18(10.59%) | 22(12.79%) |  |
|  | FL | 12(3.51%) | 5(2.94%) | 7(4.07%) |  |
|  | FLO | 7(2.05%) | 4(2.35%) | 3(1.74%) |  |
|  | FLP | 7(2.05%) | 3(1.76%) | 4(2.33%) |  |
|  | FP | 4(1.17%) | 3(1.76%) | 1(0.58%) |  |
|  | NO | 150(43.86%) | 73(42.94%) | 77(44.77%) |  |
|  | other | 9(2.63%) | 5(2.94%) | 4(2.33%) |  |
|  | P | 1(0.29%) | 1(0.59%) | 0(0%) |  |
|  | PLFE | 3(0.88%) | 0(0%) | 3(1.74%) |  |
|  | R | 1(0.29%) | 0(0%) | 1(0.58%) |  |
|  | TCX | 1(0.29%) | 0(0%) | 1(0.58%) |  |
|  | TP | 1(0.29%) | 1(0.59%) | 0(0%) |  |
|  | TS-1 | 2(0.58%) | 2(1.18%) | 0(0%) |  |
|  | TX | 2(0.58%) | 1(0.59%) | 1(0.58%) |  |
|  | unknow | 36(10.53%) | 23(13.53%) | 13(7.56%) |  |
|  | X | 8(2.34%) | 4(2.35%) | 4(2.33%) |  |
|  | XO | 2(0.58%) | 1(0.59%) | 1(0.58%) |  |
