# Supplementary material for: A novel risk model construction and immune landscape analysis of gastric cancer based on cuproptosis-related long noncoding RNAs
Source: Front Oncol. 2022 Oct 26;12:1015235. doi: 10.3389/fonc.2022.1015235 (PMC9643840; doi:10.3389/fonc.2022.1015235)
Supplement: Supplementary file 4 [file Table_2.docx]

**Supplementary Table 2** coefficients of lncRNAs in risk model

| LncRNAs | Coefficient | HR | HR.95L | HR.95H | pvalue |
| --- | --- | --- | --- | --- | --- |
| AC016737.1  AL121748.1  LINC01980  TYMSOS  AL355574.1  AL391152.1  AL353804.2  AL353796.1  AL512506.1  AC104809.2 | 0.513701417  3.000274426  0.10102639  -0.147198675  -0.276808002  1.536951075  -0.775414706  -0.627236928  -1.716860599  -0.929657971 | 1.671466553  20.09104967  1.106305837  0.86312248  0.758200056  4.650389944  0.460512764  0.534065425  0.179629192  0.394688682 | 1.252053761  3.370405044  0.968268192  0.731391826  0.598913939  2.322816868  0.187376098  0.294862804  0.053270546  0.169621049 | 2.231374183  119.7631357  1.264022318  1.018579082  0.959849633  9.310302041  1.131798604  0.967317255  0.605712705  0.918395193 | 0.000492371  0.000988031  0.137348482  0.081491297  0.021417926  0.000014279  0.091007014  0.038489428  0.005633739  0.030964734 |
