# Supplementary material for: A novel risk model construction and immune landscape analysis of gastric cancer based on cuproptosis-related long noncoding RNAs
Source: Front Oncol. 2022 Oct 26;12:1015235. doi: 10.3389/fonc.2022.1015235 (PMC9643840; doi:10.3389/fonc.2022.1015235)
Supplement: Supplementary file 5 [file Table_3.docx]

**Supplementary Table 3** siRNA/control used in this study

|  | Forward sequence (5' to 3') | Reverse sequence (5' to 3') |
| --- | --- | --- |
| Si-AL121748.1-1# | AGGAAGUUGUUGAUUUGCAGCAUAU | AUAUGCUGCAAAUCAACAACUUCCU |
| Si-AL121748.1-2#  Negative control | CGUUGUUGCUGUACCCGAUCUUGAA  GGAUAAUCUGGGAAGUGAAAUGAAA | UUCAAGAUCGGGUACAGCAACAACG  UUUCAUUUCACUUCCCAGAUUAUCC |
