# Supplementary material for: A novel risk model construction and immune landscape analysis of gastric cancer based on cuproptosis-related long noncoding RNAs
Source: Front Oncol. 2022 Oct 26;12:1015235. doi: 10.3389/fonc.2022.1015235 (PMC9643840; doi:10.3389/fonc.2022.1015235)
Supplement: Supplementary file 6 [file Table_4.docx]

**Supplementary Table 4** primers used in the study

| Name | Primer (5’-3’) |
| --- | --- |
| AL121748.1  GAPDH | F: AACAAGGCACAAAAGGGGAAAA  R: AGCAAACGCAAGGCGAAGG  F: TGCACCACCAACTGCTTAGC  R: GGCATGGACTGTGGTCATGAG |
